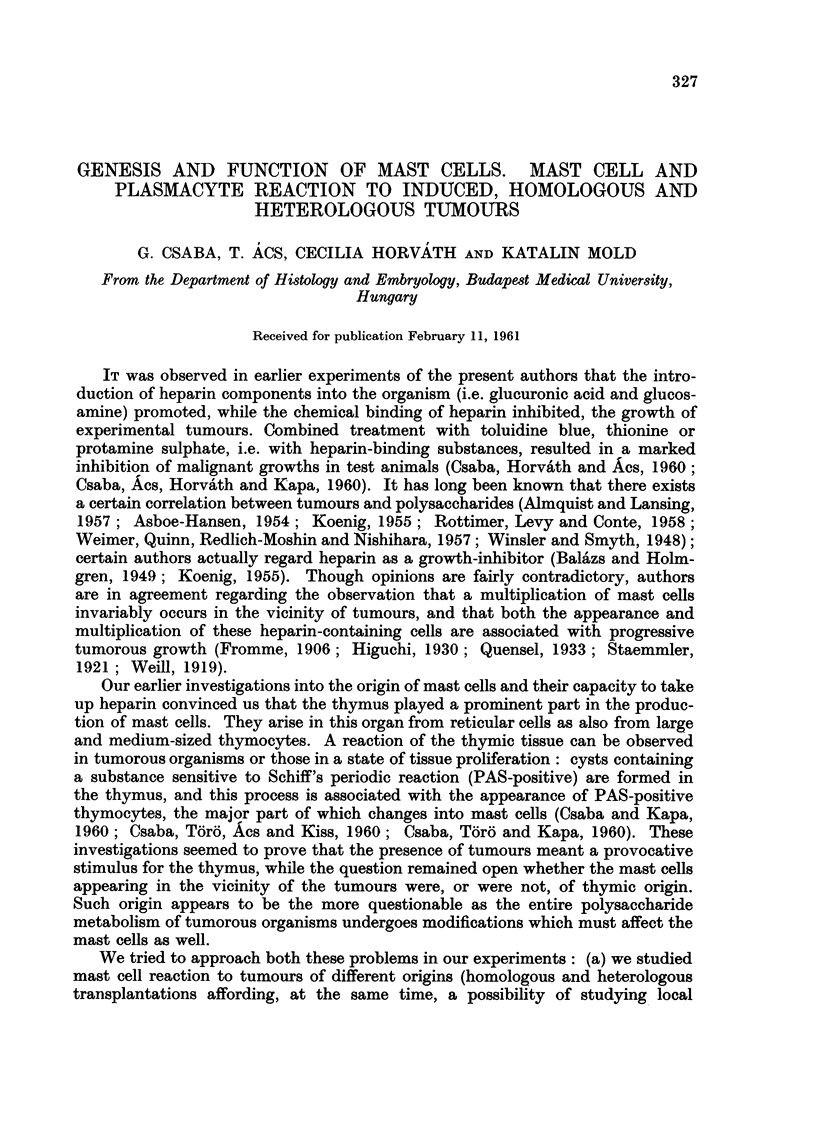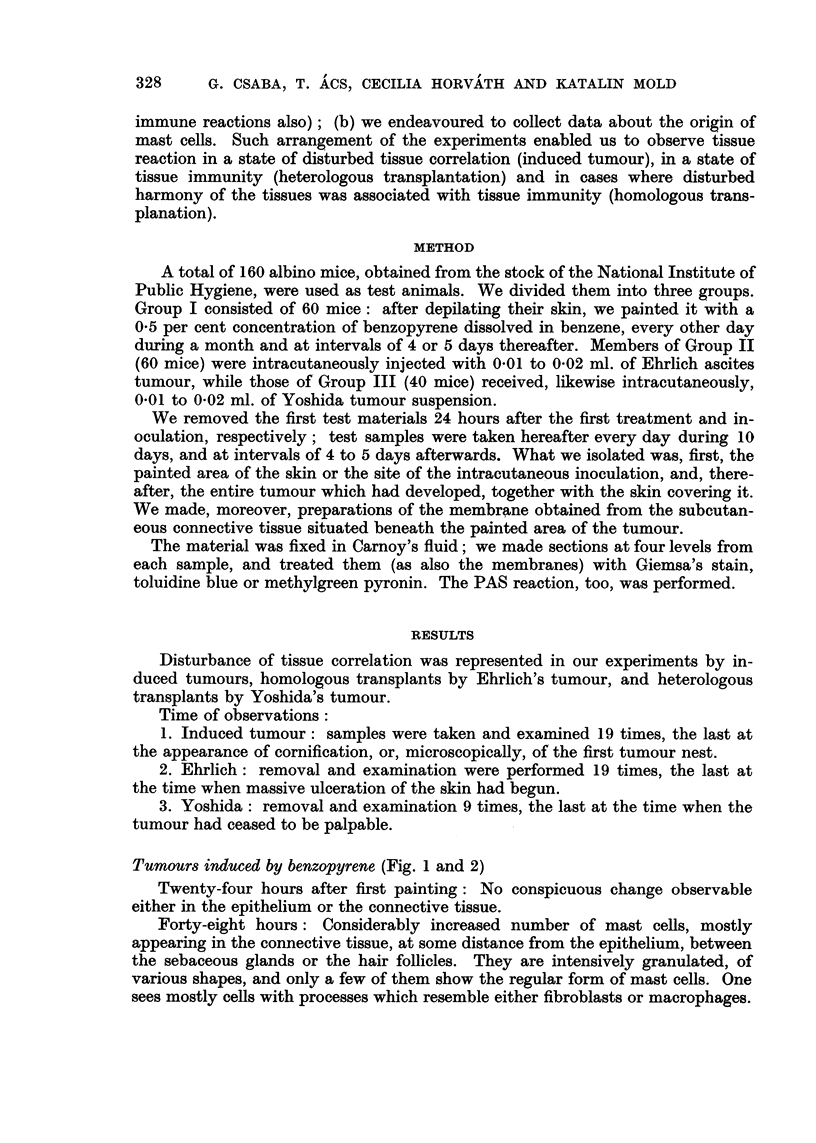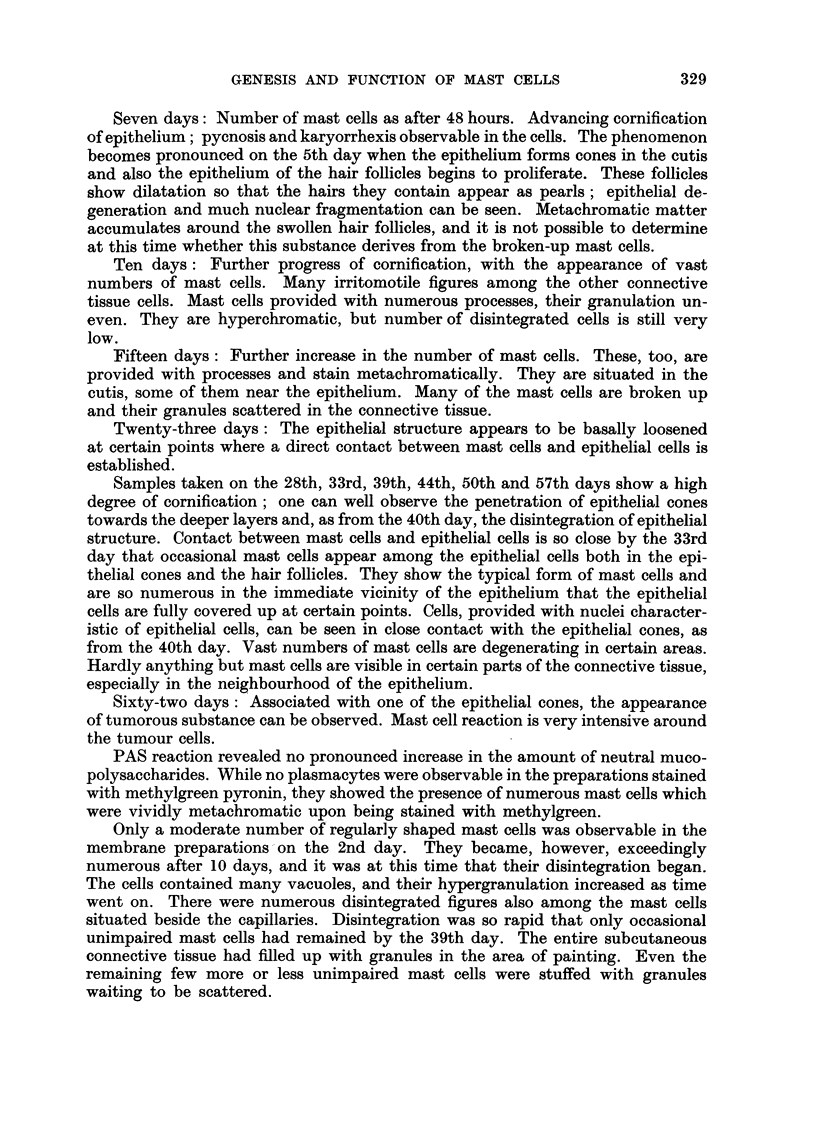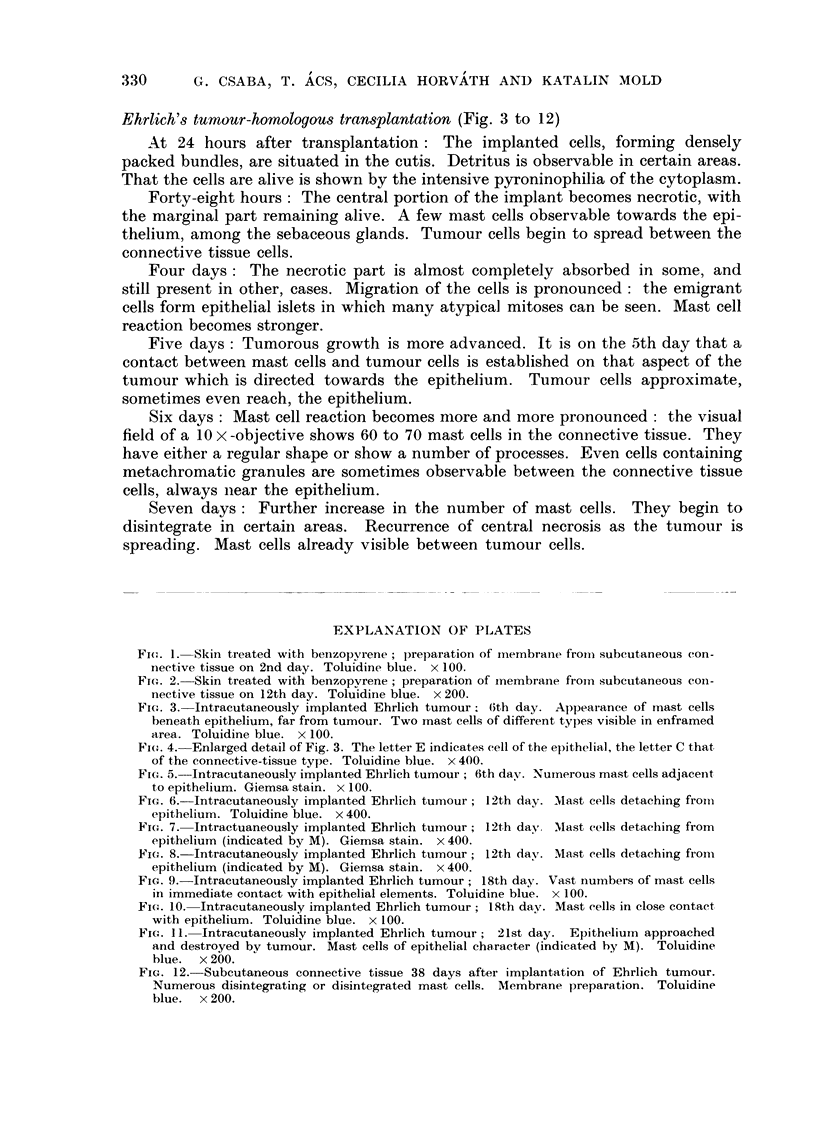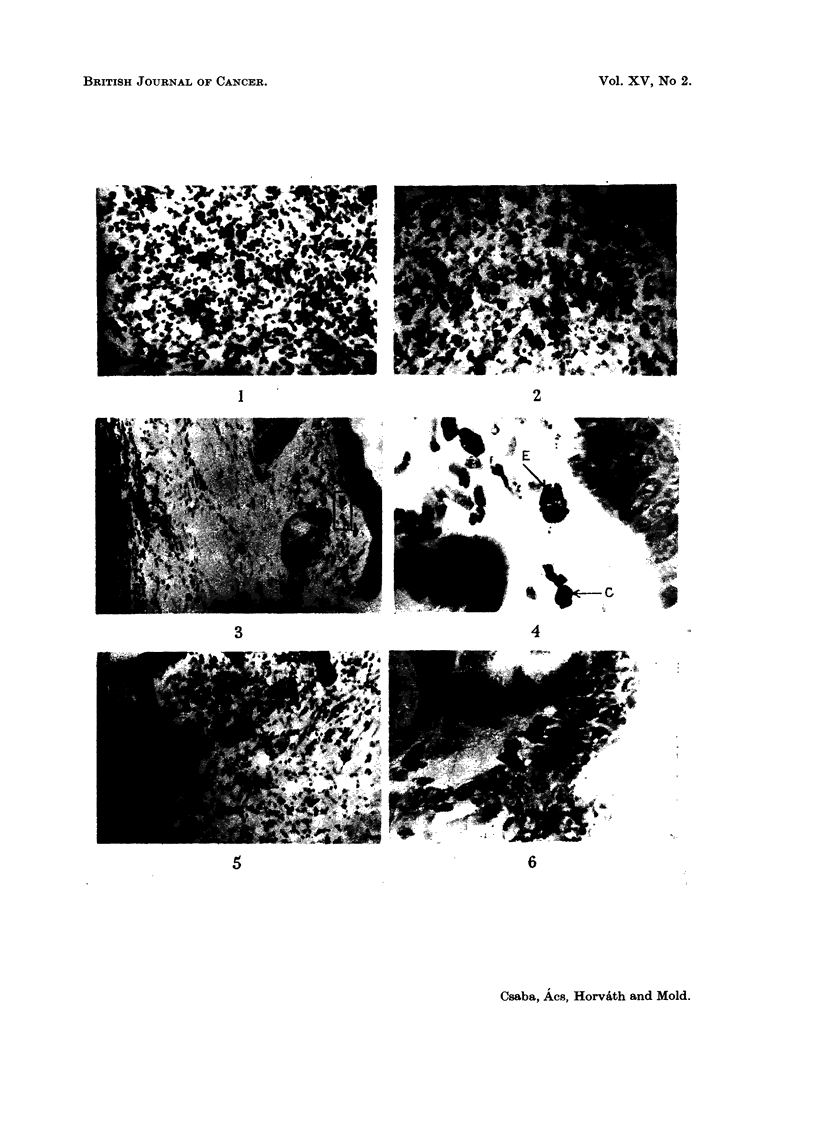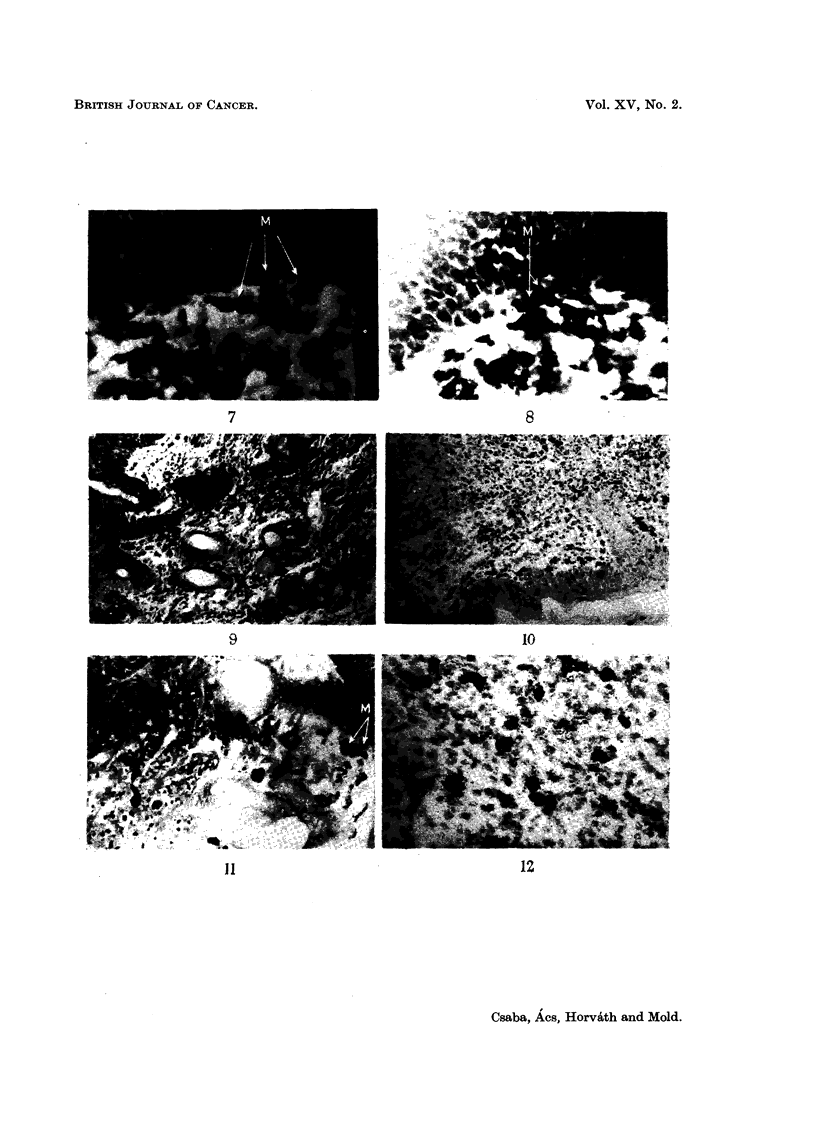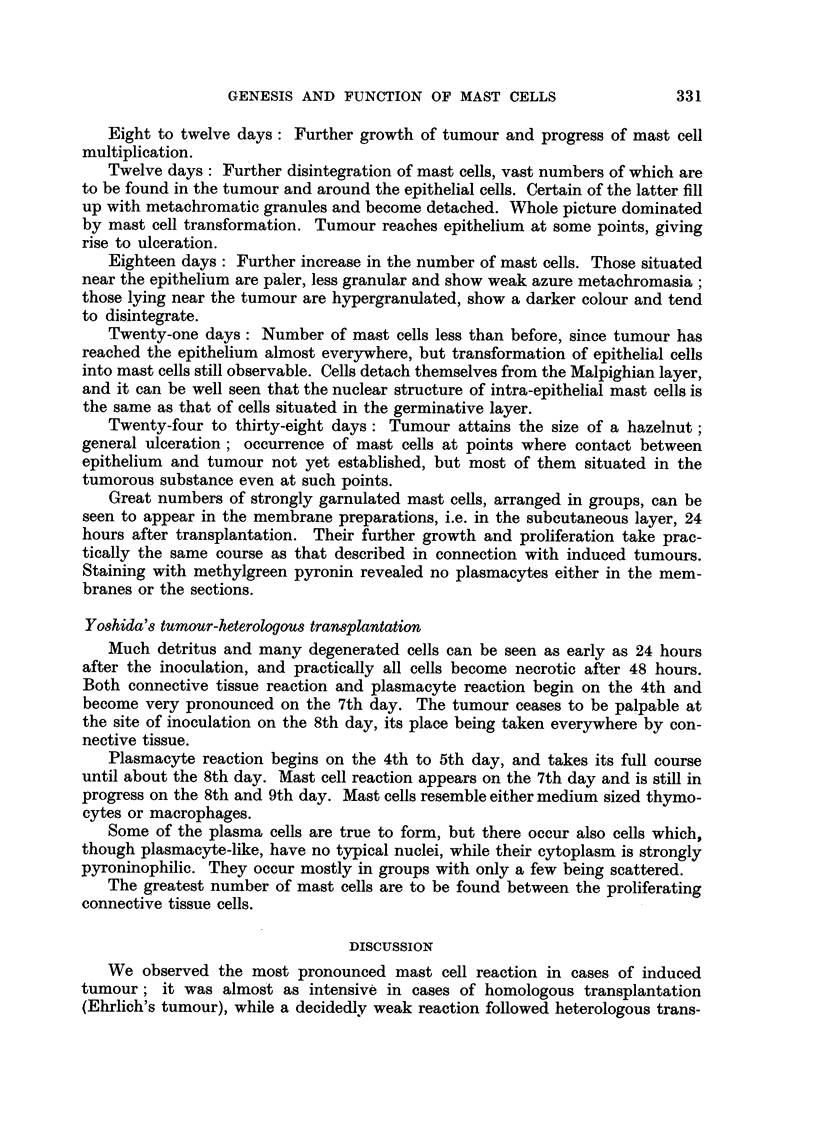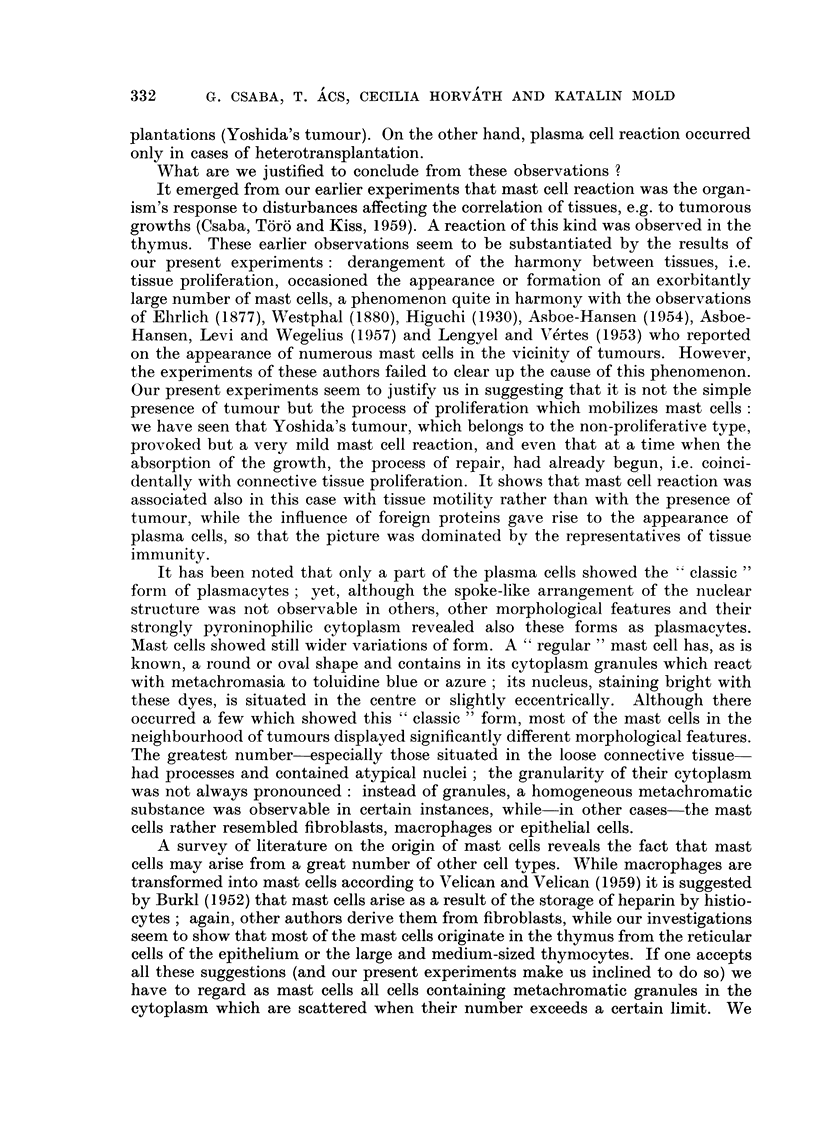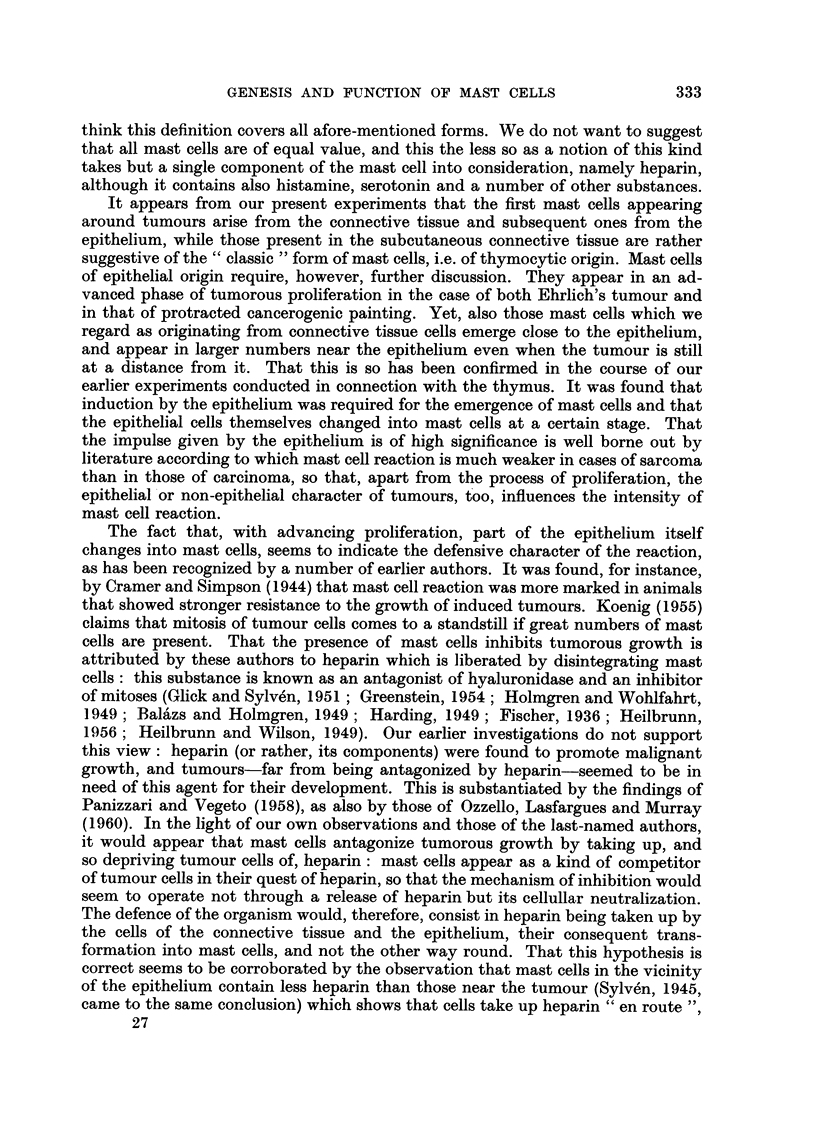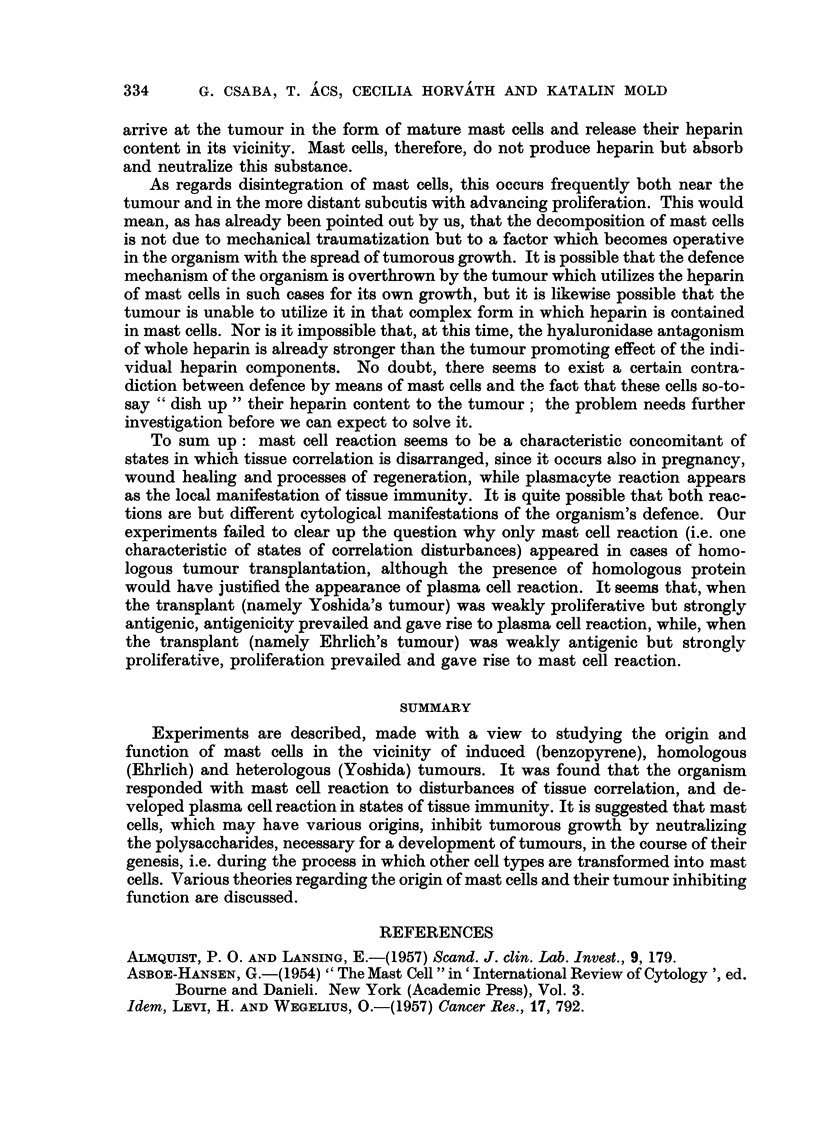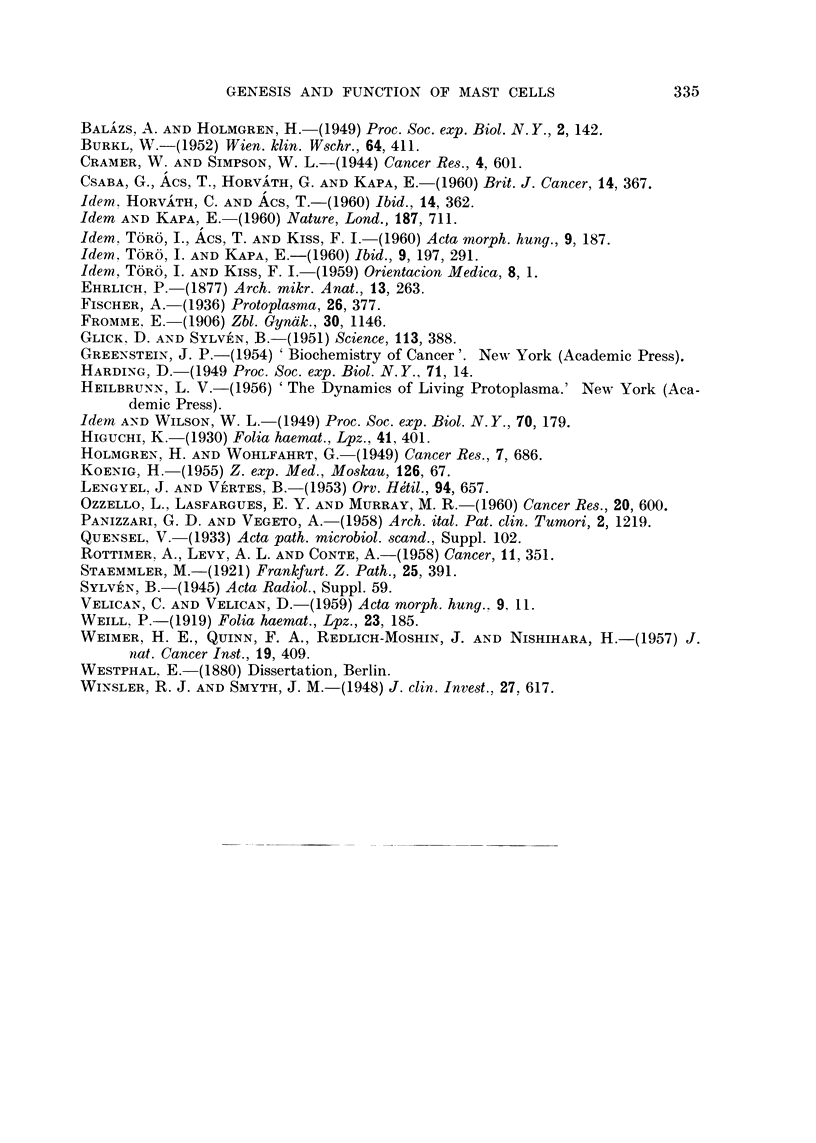# Genesis and Function of Mast Cells. Mast Cell and Plasmacyte Reaction to Induced, Homologous and Heterologous Tumours

**DOI:** 10.1038/bjc.1961.41

**Published:** 1961-06

**Authors:** G. Csaba, T. Ács, C Horváth, Katalin Mold

## Abstract

**Images:**


### GENESIS AND FUNCTION OF MAST CELLS. MAST CELL AND PLASMACYTE REACTION TO INDUCED, HOMOLOGOUS AND HETEROLOGOUS TUMOURS

G. CSABA, T. ÁCS, CECILIA HORVÁTH AND KATALIN MOLD

*From the Department of Histology and Embryology, Budapest Medical University, Hungary*

Received for publication February 11, 1961

It was observed in earlier experiments of the present authors that the introduction of heparin components into the organism (i.e. glucuronic acid and glucosamine) promoted, while the chemical binding of heparin inhibited, the growth of experimental tumours. Combined treatment with toluidine blue, thionine or protamine sulphate, i.e. with heparin-binding substances, resulted in a marked inhibition of malignant growths in test animals (Csaba, Horváth and Ács, 1960 ; Csaba, Ács, Horváth and Kapa, 1960). It has long been known that there exists a certain correlation between tumours and polysaccharides (Almquist and Lansing, 1957 ; Asboe-Hansen, 1954 ; Koenig, 1955 ; Rottimer, Levy and Conte, 1958 ; Weimer, Quinn, Redlich-Moshin and Nishihara, 1957 ; Winsler and Smyth, 1948) ; certain authors actually regard heparin as a growth-inhibitor (Balázs and Holmgren, 1949 ; Koenig, 1955). Though opinions are fairly contradictory, authors are in agreement regarding the observation that a multiplication of mast cells invariably occurs in the vicinity of tumours, and that both the appearance and multiplication of these heparin-containing cells are associated with progressive tumorous growth (Fromme, 1906 ; Higuchi, 1930 ; Quensel, 1933 ; Staemmler, 1921 ; Weill, 1919).

Our earlier investigations into the origin of mast cells and their capacity to take up heparin convinced us that the thymus played a prominent part in the production of mast cells. They arise in this organ from reticular cells as also from large and medium-sized thymocytes. A reaction of the thymic tissue can be observed in tumorous organisms or those in a state of tissue proliferation : cysts containing a substance sensitive to Schiff's periodic reaction (PAS-positive) are formed in the thymus, and this process is associated with the appearance of PAS-positive thymocytes, the major part of which changes into mast cells (Csaba and Kapa, 1960 ; Csaba, Törö, Ács and Kiss, 1960 ; Csaba, Törö and Kapa, 1960). These investigations seemed to prove that the presence of tumours meant a provocative stimulus for the thymus, while the question remained open whether the mast cells appearing in the vicinity of the tumours were, or were not, of thymic origin. Such origin appears to be the more questionable as the entire polysaccharide metabolism of tumorous organisms undergoes modifications which must affect the mast cells as well.

We tried to approach both these problems in our experiments : (a) we studied mast cell reaction to tumours of different origins (homologous and heterologous transplantations affording, at the same time, a possibility of studying local immune reactions also); (b) we endeavoured to collect data about the origin of mast cells. Such arrangement of the experiments enabled us to observe tissue reaction in a state of disturbed tissue correlation (induced tumour), in a state of tissue immunity (heterologous transplantation) and in cases where disturbed harmony of the tissues was associated with tissue immunity (homologous transplanation).

### METHOD

A total of 160 albino mice, obtained from the stock of the National Institute of Public Hygiene, were used as test animals. We divided them into three groups. Group I consisted of 60 mice: after depilating their skin, we painted it with a 0·5 per cent concentration of benzopyrene dissolved in benzene, every other day during a month and at intervals of 4 or 5 days thereafter. Members of Group II (60 mice) were intracutaneously injected with 0·01 to 0·02 ml. of Ehrlich ascites tumour, while those of Group III (40 mice) received, likewise intracutaneously, 0·01 to 0·02 ml. of Yoshida tumour suspension.

We removed the first test materials 24 hours after the first treatment and inoculation, respectively; test samples were taken hereafter every day during 10 days, and at intervals of 4 to 5 days afterwards. What we isolated was, first, the painted area of the skin or the site of the intracutaneous inoculation, and, thereafter, the entire tumour which had developed, together with the skin covering it. We made, moreover, preparations of the membrane obtained from the subcutaneous connective tissue situated beneath the painted area of the tumour.

The material was fixed in Carnoy's fluid; we made sections at four levels from each sample, and treated them (as also the membranes) with Giemsa's stain, toluidine blue or methylgreen pyronin. The PAS reaction, too, was performed.

### RESULTS

Disturbance of tissue correlation was represented in our experiments by induced tumours, homologous transplants by Ehrlich's tumour, and heterologous transplants by Yoshida's tumour.

Time of observations:

1. Induced tumour: samples were taken and examined 19 times, the last at the appearance of cornification, or, microscopically, of the first tumour nest.

2. Ehrlich: removal and examination were performed 19 times, the last at the time when massive ulceration of the skin had begun.

3. Yoshida: removal and examination 9 times, the last at the time when the tumour had ceased to be palpable.

*Tumours induced by benzopyrene* (Fig. 1 and 2)

Twenty-four hours after first painting: No conspicuous change observable either in the epithelium or the connective tissue.

Forty-eight hours: Considerably increased number of mast cells, mostly appearing in the connective tissue, at some distance from the epithelium, between the sebaceous glands or the hair follicles. They are intensively granulated, of various shapes, and only a few of them show the regular form of mast cells. One sees mostly cells with processes which resemble either fibroblasts or macrophages.

Seven days: Number of mast cells as after 48 hours. Advancing cornification of epithelium; pycnosis and karyorrhexis observable in the cells. The phenomenon becomes pronounced on the 5th day when the epithelium forms cones in the cutis and also the epithelium of the hair follicles begins to proliferate. These follicles show dilatation so that the hairs they contain appear as pearls; epithelial degeneration and much nuclear fragmentation can be seen. Metachromatic matter accumulates around the swollen hair follicles, and it is not possible to determine at this time whether this substance derives from the broken-up mast cells.

Ten days: Further progress of cornification, with the appearance of vast numbers of mast cells. Many irritomotile figures among the other connective tissue cells. Mast cells provided with numerous processes, their granulation uneven. They are hyperchromatic, but number of disintegrated cells is still very low.

Fifteen days: Further increase in the number of mast cells. These, too, are provided with processes and stain metachromatically. They are situated in the cutis, some of them near the epithelium. Many of the mast cells are broken up and their granules scattered in the connective tissue.

Twenty-three days: The epithelial structure appears to be basally loosened at certain points where a direct contact between mast cells and epithelial cells is established.

Samples taken on the 28th, 33rd, 39th, 44th, 50th and 57th days show a high degree of cornification; one can well observe the penetration of epithelial cones towards the deeper layers and, as from the 40th day, the disintegration of epithelial structure. Contact between mast cells and epithelial cells is so close by the 33rd day that occasional mast cells appear among the epithelial cells both in the epithelial cones and the hair follicles. They show the typical form of mast cells and are so numerous in the immediate vicinity of the epithelium that the epithelial cells are fully covered up at certain points. Cells, provided with nuclei characteristic of epithelial cells, can be seen in close contact with the epithelial cones, as from the 40th day. Vast numbers of mast cells are degenerating in certain areas. Hardly anything but mast cells are visible in certain parts of the connective tissue, especially in the neighbourhood of the epithelium.

Sixty-two days: Associated with one of the epithelial cones, the appearance of tumorous substance can be observed. Mast cell reaction is very intensive around the tumour cells.

PAS reaction revealed no pronounced increase in the amount of neutral mucopolysaccharides. While no plasmacytes were observable in the preparations stained with methylgreen pyronin, they showed the presence of numerous mast cells which were vividly metachromatic upon being stained with methylgreen.

Only a moderate number of regularly shaped mast cells was observable in the membrane preparations on the 2nd day. They became, however, exceedingly numerous after 10 days, and it was at this time that their disintegration began. The cells contained many vacuoles, and their hypergranulation increased as time went on. There were numerous disintegrated figures also among the mast cells situated beside the capillaries. Disintegration was so rapid that only occasional unimpaired mast cells had remained by the 39th day. The entire subcutaneous connective tissue had filled up with granules in the area of painting. Even the remaining few more or less unimpaired mast cells were stuffed with granules waiting to be scattered.

*Ehrlich's tumour-homologous transplantation* (Fig. 3 to 12)

At 24 hours after transplantation: The implanted cells, forming densely packed bundles, are situated in the cutis. Detritus is observable in certain areas. That the cells are alive is shown by the intensive pyroninophilia of the cytoplasm.

Forty-eight hours: The central portion of the implant becomes necrotic, with the marginal part remaining alive. A few mast cells observable towards the epithelium, among the sebaceous glands. Tumour cells begin to spread between the connective tissue cells.

Four days: The necrotic part is almost completely absorbed in some, and still present in other, cases. Migration of the cells is pronounced: the emigrant cells form epithelial islets in which many atypical mitoses can be seen. Mast cell reaction becomes stronger.

Five days: Tumorous growth is more advanced. It is on the 5th day that a contact between mast cells and tumour cells is established on that aspect of the tumour which is directed towards the epithelium. Tumour cells approximate, sometimes even reach, the epithelium.

Six days: Mast cell reaction becomes more and more pronounced: the visual field of a 10×-objective shows 60 to 70 mast cells in the connective tissue. They have either a regular shape or show a number of processes. Even cells containing metachromatic granules are sometimes observable between the connective tissue cells, always near the epithelium.

Seven days: Further increase in the number of mast cells. They begin to disintegrate in certain areas. Recurrence of central necrosis as the tumour is spreading. Mast cells already visible between tumour cells.

---

### EXPLANATION OF PLATES

FIG. 1.—Skin treated with benzopyrene; preparation of membrane from subcutaneous connective tissue on 2nd day. Toluidine blue. ×100.

FIG. 2.—Skin treated with benzopyrene; preparation of membrane from subcutaneous connective tissue on 12th day. Toluidine blue. ×200.

FIG. 3.—Intracutaneously implanted Ehrlich tumour; 6th day. Appearance of mast cells beneath epithelium, far from tumour. Two mast cells of different types visible in enframed area. Toluidine blue. ×100.

FIG. 4.—Enlarged detail of Fig. 3. The letter E indicates cell of the epithelial, the letter C that of the connective-tissue type. Toluidine blue. ×400.

FIG. 5.—Intracutaneously implanted Ehrlich tumour; 6th day. Numerous mast cells adjacent to epithelium. Giemsa stain. ×100.

FIG. 6.—Intracutaneously implanted Ehrlich tumour; 12th day. Mast cells detaching from epithelium. Toluidine blue. ×400.

FIG. 7.—Intractuaneously implanted Ehrlich tumour; 12th day. Mast cells detaching from epithelium (indicated by M). Giemsa stain. ×400.

FIG. 8.—Intracutaneously implanted Ehrlich tumour; 12th day. Mast cells detaching from epithelium (indicated by M). Giemsa stain. ×400.

FIG. 9.—Intracutaneously implanted Ehrlich tumour; 18th day. Vast numbers of mast cells in immediate contact with epithelial elements. Toluidine blue. ×100.

FIG. 10.—Intracutaneously implanted Ehrlich tumour; 18th day. Mast cells in close contact with epithelium. Toluidine blue. ×100.

FIG. 11.—Intracutaneously implanted Ehrlich tumour; 21st day. Epithelium approached and destroyed by tumour. Mast cells of epithelial character (indicated by M). Toluidine blue. ×200.

FIG. 12.—Subcutaneous connective tissue 38 days after implantation of Ehrlich tumour. Numerous disintegrating or disintegrated mast cells. Membrane preparation. Toluidine blue. ×200.

1

2

3

4

5

6

Csaba, Ács, Horváth and Mold.

7

8

9

10

11

12

Eight to twelve days : Further growth of tumour and progress of mast cell multiplication.

Twelve days : Further disintegration of mast cells, vast numbers of which are to be found in the tumour and around the epithelial cells. Certain of the latter fill up with metachromatic granules and become detached. Whole picture dominated by mast cell transformation. Tumour reaches epithelium at some points, giving rise to ulceration.

Eighteen days : Further increase in the number of mast cells. Those situated near the epithelium are paler, less granular and show weak azure metachromasia ; those lying near the tumour are hypergranulated, show a darker colour and tend to disintegrate.

Twenty-one days : Number of mast cells less than before, since tumour has reached the epithelium almost everywhere, but transformation of epithelial cells into mast cells still observable. Cells detach themselves from the Malpighian layer, and it can be well seen that the nuclear structure of intra-epithelial mast cells is the same as that of cells situated in the germinative layer.

Twenty-four to thirty-eight days : Tumour attains the size of a hazelnut ; general ulceration ; occurrence of mast cells at points where contact between epithelium and tumour not yet established, but most of them situated in the tumorous substance even at such points.

Great numbers of strongly garnulated mast cells, arranged in groups, can be seen to appear in the membrane preparations, i.e. in the subcutaneous layer, 24 hours after transplantation. Their further growth and proliferation take practically the same course as that described in connection with induced tumours. Staining with methylgreen pyronin revealed no plasmacytes either in the membranes or the sections.

*Yoshida's tumour-heterologous transplantation*

Much detritus and many degenerated cells can be seen as early as 24 hours after the inoculation, and practically all cells become necrotic after 48 hours. Both connective tissue reaction and plasmacyte reaction begin on the 4th and become very pronounced on the 7th day. The tumour ceases to be palpable at the site of inoculation on the 8th day, its place being taken everywhere by connective tissue.

Plasmacyte reaction begins on the 4th to 5th day, and takes its full course until about the 8th day. Mast cell reaction appears on the 7th day and is still in progress on the 8th and 9th day. Mast cells resemble either medium sized thymocytes or macrophages.

Some of the plasma cells are true to form, but there occur also cells which, though plasmacyte-like, have no typical nuclei, while their cytoplasm is strongly pyroninophilic. They occur mostly in groups with only a few being scattered.

The greatest number of mast cells are to be found between the proliferating connective tissue cells.

### DISCUSSION

We observed the most pronounced mast cell reaction in cases of induced tumour ; it was almost as intensive in cases of homologous transplantation (Ehrlich's tumour), while a decidedly weak reaction followed heterologous transplantations (Yoshida's tumour). On the other hand, plasma cell reaction occurred only in cases of heterotransplantation.

What are we justified to conclude from these observations ?

It emerged from our earlier experiments that mast cell reaction was the organism's response to disturbances affecting the correlation of tissues, e.g. to tumorous growths (Csaba, Törö and Kiss, 1959). A reaction of this kind was observed in the thymus. These earlier observations seem to be substantiated by the results of our present experiments : derangement of the harmony between tissues, i.e. tissue proliferation, occasioned the appearance or formation of an exorbitantly large number of mast cells, a phenomenon quite in harmony with the observations of Ehrlich (1877), Westphal (1880), Higuchi (1930), Asboe-Hansen (1954), Asboe-Hansen, Levi and Wegelius (1957) and Lengyel and Vértes (1953) who reported on the appearance of numerous mast cells in the vicinity of tumours. However, the experiments of these authors failed to clear up the cause of this phenomenon. Our present experiments seem to justify us in suggesting that it is not the simple presence of tumour but the process of proliferation which mobilizes mast cells : we have seen that Yoshida's tumour, which belongs to the non-proliferative type, provoked but a very mild mast cell reaction, and even that at a time when the absorption of the growth, the process of repair, had already begun, i.e. coincidentally with connective tissue proliferation. It shows that mast cell reaction was associated also in this case with tissue motility rather than with the presence of tumour, while the influence of foreign proteins gave rise to the appearance of plasma cells, so that the picture was dominated by the representatives of tissue immunity.

It has been noted that only a part of the plasma cells showed the " classic " form of plasmacytes ; yet, although the spoke-like arrangement of the nuclear structure was not observable in others, other morphological features and their strongly pyroninophilic cytoplasm revealed also these forms as plasmacytes. Mast cells showed still wider variations of form. A " regular " mast cell has, as is known, a round or oval shape and contains in its cytoplasm granules which react with metachromasia to toluidine blue or azure ; its nucleus, staining bright with these dyes, is situated in the centre or slightly eccentrically. Although there occurred a few which showed this " classic " form, most of the mast cells in the neighbourhood of tumours displayed significantly different morphological features. The greatest number—especially those situated in the loose connective tissue—had processes and contained atypical nuclei ; the granularity of their cytoplasm was not always pronounced : instead of granules, a homogeneous metachromatic substance was observable in certain instances, while—in other cases—the mast cells rather resembled fibroblasts, macrophages or epithelial cells.

A survey of literature on the origin of mast cells reveals the fact that mast cells may arise from a great number of other cell types. While macrophages are transformed into mast cells according to Velican and Velican (1959) it is suggested by Burkl (1952) that mast cells arise as a result of the storage of heparin by histiocytes ; again, other authors derive them from fibroblasts, while our investigations seem to show that most of the mast cells originate in the thymus from the reticular cells of the epithelium or the large and medium-sized thymocytes. If one accepts all these suggestions (and our present experiments make us inclined to do so) we have to regard as mast cells all cells containing metachromatic granules in the cytoplasm which are scattered when their number exceeds a certain limit. We think this definition covers all afore-mentioned forms. We do not want to suggest that all mast cells are of equal value, and this the less so as a notion of this kind takes but a single component of the mast cell into consideration, namely heparin, although it contains also histamine, serotonin and a number of other substances.

It appears from our present experiments that the first mast cells appearing around tumours arise from the connective tissue and subsequent ones from the epithelium, while those present in the subcutaneous connective tissue are rather suggestive of the " classic " form of mast cells, i.e. of thymocytic origin. Mast cells of epithelial origin require, however, further discussion. They appear in an advanced phase of tumorous proliferation in the case of both Ehrlich's tumour and in that of protracted cancerogenic painting. Yet, also those mast cells which we regard as originating from connective tissue cells emerge close to the epithelium, and appear in larger numbers near the epithelium even when the tumour is still at a distance from it. That this is so has been confirmed in the course of our earlier experiments conducted in connection with the thymus. It was found that induction by the epithelium was required for the emergence of mast cells and that the epithelial cells themselves changed into mast cells at a certain stage. That the impulse given by the epithelium is of high significance is well borne out by literature according to which mast cell reaction is much weaker in cases of sarcoma than in those of carcinoma, so that, apart from the process of proliferation, the epithelial or non-epithelial character of tumours, too, influences the intensity of mast cell reaction.

The fact that, with advancing proliferation, part of the epithelium itself changes into mast cells, seems to indicate the defensive character of the reaction, as has been recognized by a number of earlier authors. It was found, for instance, by Cramer and Simpson (1944) that mast cell reaction was more marked in animals that showed stronger resistance to the growth of induced tumours. Koenig (1955) claims that mitosis of tumour cells comes to a standstill if great numbers of mast cells are present. That the presence of mast cells inhibits tumorous growth is attributed by these authors to heparin which is liberated by disintegrating mast cells : this substance is known as an antagonist of hyaluronidase and an inhibitor of mitoses (Glick and Sylvén, 1951 ; Greenstein, 1954 ; Holmgren and Wohlfahrt, 1949 ; Balázs and Holmgren, 1949 ; Harding, 1949 ; Fischer, 1936 ; Heilbrunn, 1956 ; Heilbrunn and Wilson, 1949). Our earlier investigations do not support this view : heparin (or rather, its components) were found to promote malignant growth, and tumours—far from being antagonized by heparin—seemed to be in need of this agent for their development. This is substantiated by the findings of Panizzari and Vegeto (1958), as also by those of Ozzello, Lasfargues and Murray (1960). In the light of our own observations and those of the last-named authors, it would appear that mast cells antagonize tumorous growth by taking up, and so depriving tumour cells of, heparin : mast cells appear as a kind of competitor of tumour cells in their quest of heparin, so that the mechanism of inhibition would seem to operate not through a release of heparin but its cellullar neutralization. The defence of the organism would, therefore, consist in heparin being taken up by the cells of the connective tissue and the epithelium, their consequent transformation into mast cells, and not the other way round. That this hypothesis is correct seems to be corroborated by the observation that mast cells in the vicinity of the epithelium contain less heparin than those near the tumour (Sylvén, 1945, came to the same conclusion) which shows that cells take up heparin " en route ", arrive at the tumour in the form of mature mast cells and release their heparin content in its vicinity. Mast cells, therefore, do not produce heparin but absorb and neutralize this substance.

As regards disintegration of mast cells, this occurs frequently both near the tumour and in the more distant subcutis with advancing proliferation. This would mean, as has already been pointed out by us, that the decomposition of mast cells is not due to mechanical traumatization but to a factor which becomes operative in the organism with the spread of tumorous growth. It is possible that the defence mechanism of the organism is overthrown by the tumour which utilizes the heparin of mast cells in such cases for its own growth, but it is likewise possible that the tumour is unable to utilize it in that complex form in which heparin is contained in mast cells. Nor is it impossible that, at this time, the hyaluronidase antagonism of whole heparin is already stronger than the tumour promoting effect of the individual heparin components. No doubt, there seems to exist a certain contradiction between defence by means of mast cells and the fact that these cells so-to-say " dish up " their heparin content to the tumour ; the problem needs further investigation before we can expect to solve it.

To sum up : mast cell reaction seems to be a characteristic concomitant of states in which tissue correlation is disarranged, since it occurs also in pregnancy, wound healing and processes of regeneration, while plasmacyte reaction appears as the local manifestation of tissue immunity. It is quite possible that both reactions are but different cytological manifestations of the organism's defence. Our experiments failed to clear up the question why only mast cell reaction (i.e. one characteristic of states of correlation disturbances) appeared in cases of homologous tumour transplantation, although the presence of homologous protein would have justified the appearance of plasma cell reaction. It seems that, when the transplant (namely Yoshida's tumour) was weakly proliferative but strongly antigenic, antigenicity prevailed and gave rise to plasma cell reaction, while, when the transplant (namely Ehrlich's tumour) was weakly antigenic but strongly proliferative, proliferation prevailed and gave rise to mast cell reaction.

SUMMARY

Experiments are described, made with a view to studying the origin and function of mast cells in the vicinity of induced (benzopyrene), homologous (Ehrlich) and heterologous (Yoshida) tumours. It was found that the organism responded with mast cell reaction to disturbances of tissue correlation, and developed plasma cell reaction in states of tissue immunity. It is suggested that mast cells, which may have various origins, inhibit tumorous growth by neutralizing the polysaccharides, necessary for a development of tumours, in the course of their genesis, i.e. during the process in which other cell types are transformed into mast cells. Various theories regarding the origin of mast cells and their tumour inhibiting function are discussed.

### REFERENCES

ALMQUIST, P. O. AND LANSING, E.—(1957) *Scand. J. clin. Lab. Invest.*, **9**, 179.

ASBOE-HANSEN, G.—(1954) " The Mast Cell " in ' International Review of Cytology ', ed. Bourne and Danieli. New York (Academic Press), Vol. 3.

*Idem*, LEVI, H. AND WEGELIUS, O.—(1957) *Cancer Res.*, **17**, 792.

Balázs, A. and Holmgren, H.—(1949) *Proc. Soc. exp. Biol. N.Y.*, **2**, 142.
Burkl, W.—(1952) *Wien. klin. Wschr.*, **64**, 411.
Cramer, W. and Simpson, W. L.—(1944) *Cancer Res.*, **4**, 601.
Csaba, G., Ács, T., Horváth, G. and Kapa, E.—(1960) *Brit. J. Cancer*, **14**, 367.
*Idem*, Horváth, C. and Ács, T.—(1960) *Ibid.*, **14**, 362.
*Idem* and Kapa, E.—(1960) *Nature, Lond.*, **187**, 711.
*Idem*, Törö, I., Ács, T. and Kiss, F. I.—(1960) *Acta morph. hung.*, **9**, 187.
*Idem*, Törö, I. and Kapa, E.—(1960) *Ibid.*, **9**, 197, 291.
*Idem*, Törö, I. and Kiss, F. I.—(1959) *Orientacion Medica*, **8**, 1.
Ehrlich, P.—(1877) *Arch. mikr. Anat.*, **13**, 263.
Fischer, A.—(1936) *Protoplasma*, **26**, 377.
Fromme, E.—(1906) *Zbl. Gynäk.*, **30**, 1146.
Glick, D. and Sylvén, B.—(1951) *Science*, **113**, 388.
Greenstein, J. P.—(1954) 'Biochemistry of Cancer'. New York (Academic Press).
Harding, D.—(1949 *Proc. Soc. exp. Biol. N.Y.*, **71**, 14.
Heilbrunn, L. V.—(1956) 'The Dynamics of Living Protoplasma.' New York (Academic Press).
*Idem* and Wilson, W. L.—(1949) *Proc. Soc. exp. Biol. N.Y.*, **70**, 179.
Higuchi, K.—(1930) *Folia haemat., Lpz.*, **41**, 401.
Holmgren, H. and Wohlfahrt, G.—(1949) *Cancer Res.*, **7**, 686.
Koenig, H.—(1955) *Z. exp. Med., Moskau*, **126**, 67.
Lengyel, J. and Vértes, B.—(1953) *Orv. Hétil.*, **94**, 657.
Ozzello, L., Lasfargues, E. Y. and Murray, M. R.—(1960) *Cancer Res.*, **20**, 600.
Panizzari, G. D. and Vegeto, A.—(1958) *Arch. ital. Pat. clin. Tumori*, **2**, 1219.
Quensel, V.—(1933) *Acta path. microbiol. scand.*, Suppl. 102.
Rottimer, A., Levy, A. L. and Conte, A.—(1958) *Cancer*, **11**, 351.
Staemmler, M.—(1921) *Frankfurt. Z. Path.*, **25**, 391.
Sylvén, B.—(1945) *Acta Radiol.*, Suppl. 59.
Velican, C. and Velican, D.—(1959) *Acta morph. hung.*, **9**, 11.
Weill, P.—(1919) *Folia haemat., Lpz.*, **23**, 185.
Weimer, H. E., Quinn, F. A., Redlich-Moshin, J. and Nishihara, H.—(1957) *J. nat. Cancer Inst.*, **19**, 409.
Westphal, E.—(1880) Dissertation, Berlin.
Winsler, R. J. and Smyth, J. M.—(1948) *J. clin. Invest.*, **27**, 617.